# A novel mechanical cleavage method for synthesizing few-layer graphenes

**DOI:** 10.1186/1556-276X-6-95

**Published:** 2011-01-19

**Authors:** Buddhika Jayasena, Sathyan Subbiah

**Affiliations:** 1 School of Mechanical and Aerospace Engineering, Nanyang Technological University, 50 Nanyang Avenue, 639798 Singapore

## Abstract

A novel method to synthesize few layer graphene from bulk graphite by mechanical cleavage is presented here. The method involves the use of an ultrasharp single crystal diamond wedge to cleave a highly ordered pyrolytic graphite sample to generate the graphene layers. Cleaving is aided by the use of ultrasonic oscillations along the wedge. Characterization of the obtained layers shows that the process is able to synthesize graphene layers with an area of a few micrometers. Application of oscillation enhances the quality of the layers produced with the layers having a reduced crystallite size as determined from the Raman spectrum. Interesting edge structures are observed that needs further investigation.

## Introduction

There is an urgent need to develop a large-scale method to manufacture graphene reliably for various promising applications being developed [1]. These applications rely largely on the unique properties of graphene [2,3] and the properties are strongly affected by the method of synthesis [4]. While several laboratory methods to synthesize graphene have been developed and reported, the suitability of these methods to large-scale manufacturing remains to be proven. These methods can be broadly classified as epitaxial growth, colloidal suspension, unconventional methods, and exfoliation. In the epitaxial growth method graphene can be grown on top of either metallic or insulator substrates using physical and chemical vapour deposition methods [2,4,5]. In the colloidal suspension method, a combination of aqueous or organic solvent with an initial raw material such as graphite oxide is used [2]. There are also several unconventional methods such as unzipping carbon nanotubes (CNT), arc discharge, and detonation using chemicals that have been explored for graphene manufacturing. The unzipping of CNT can be categorized as an oxidizing method involving insertion of metal atoms with ammonia using thermal treatment, plasma cutting after embedding in polymer, and catalytic microwave cutting [6]. The arc discharge method involves the use of a high-current arc discharge between a graphite anode and graphite cathode in a chamber filled with hydrogen and helium gas [7]. In the detonation method, a mixture of natural graphite, nitric acid, and CH_3_NO_2 _is exploded in a vessel and graphene detected in the soot obtained [8]. All these methods suffer from various limitations such as poor yield, use of special hazardous chemicals, and contamination of graphene with impurities or functional groups, and long processing time. The exfoliation method, the method of interest in this paper, essentially involves separation of graphene layers from bulk graphite; this technique can be further classified into thermal, chemical, or mechanical methods.

In thermal exfoliation, graphite (natural or graphite oxide) is used as the starting material and the process comprises of three steps: oxidization, thermal expansion/exfoliation and centrifugation, and ultrasonication [9]. Chemical exfoliation is carried out at high temperatures and involves several process steps and chemicals [10]. The devices can be fabricated on several surfaces, and deposition of graphene from solution is the main merit of this method. Mechanical exfoliation, the main focus of this paper, is another laboratory-based method for graphene sample preparation. The scotch tape method is the popular method of mechanical cleavage [11] that has been explored for separation of graphene. Repeated peeling is needed to achieve single layer graphene and it is difficult to predict the number of peelings required. Another micromechanical cleaving method reported by Ruoff et al [12] involves the use of an atomic force microscope (AFM) tip along with an array of highly ordered pyrolytic graphite (HOPG) mesas made from oxygen-plasma etching method. The HOPG islands were transferred to a SiO_2_/Si substrate using hydrofluoric acid. It is then manipulated using an AFM tip to obtain multiple layers of HOPG. A variation of this method involves gluing a block of prepared graphite to an AFM tip and scratched on Si substrates [13]. In general, it is difficult to control the separation and number of graphene layers generated using these mechanical methods. In this context, there is further scope in exploring other mechanical exfoliation techniques for graphene synthesis with potential for low chemical usage and better process controllability. Application areas where ultrathin sectioning is routinely performed offer some ideas for mechanical exfoliation of layers from a bulk substrate.

Use of an ultrasharp wedge as a sectioning method has been used in biological sample preparation and ultrathin samples (as thin as 40 nm) are generated with either glass or diamond wedges [14]. Mica, a layered material, was cleaved using a glass wedge proving the possibility of layer separation as early as in 1930 [15]. Brittle and hard materials such as germanium have also been sectioned to nanometer-scale thickness using this technique [16]. Reproducibility of section thickness, chemical inertness, and durability of the diamond wedge are the main advantages of this technique. Thus, there is potential in exploring the use of this technique in graphene synthesis as well.

Here, we adopt this sectioning technique to develop a novel mechanical exfoliation method to synthesize few layer graphene from bulk graphite. The method uses an ultrasharp single crystal diamond wedge to exfoliate a highly ordered pyrolytic graphite sample and generate the graphene layers. We test the effect of high-frequency oscillations applied along the wedge, which will enable a smooth sliding motion of the cleaved layers over the diamond wedge surface leading to better quality layers. The thickness of the layers obtained is analyzed using AFM and transmission electron microscopy (TEM) to study the layer structure and the edges in detail. The effect of applied oscillations is studied by calculating the crystallite size from Raman spectroscopic analysis.

### Experimental setup and characterization methods

**Figure 1:**
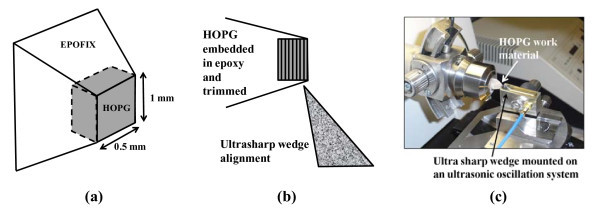
**HOPG, SPI grade ZYH**. **(a) **HOPG mounted in epofix and trimmed to pyramid shape. **(b) **Setup showing wedge alignment with HOPG layers. **(c) **Actual experimental setup.

HOPG, SPI grade ZYH, with dimensions of 2 × 12 × 12 mm, is used as the starting substrate material. The HOPG is first cut into small pieces of size 1 × 1 × 2 mm using a sharp blade and then embedded into an epofix embedding medium. It is then trimmed as shown in Figure 1a into a pyramid shape using a trimming machine so as to make it ready for cleaving. The ultrasharp wedge used for sectioning is made of a single crystal diamond with sharpness less than 20 Å and has an included angle of 35°. The diamond wedge is mounted on an ultrasonic oscillation system capable of providing tunable frequencies in the 10-kHz range (25-45 kHz) with an amplitude of vibration of a few tens of nanometers (set as a voltage value in the range 0-30 V). The diamond wedge mounted on the oscillation system is aligned carefully with respect to the HOPG mount (Figure 1b). The HOPG and the diamond wedge system are mounted on two different high-precision slide systems on a Leica Ultracut system (Figure 1c). The ultrasharp wedge is held fixed while the work material is fed slowly downwards at a controlled speed (0.6 mm/s) towards the wedge. The overlap between the diamond wedge and the HOPG surface is set to 40 nm. A tool setting angle of 6°, frequency of vibration of 0 kHz (no oscillation) and 33.1 kHz, and voltage of 2.1 V are used as process parameters. The cleaved layers slide off the diamond wedge surface, are floated on to a water bath arrangement, collected onto a loop and then transferred to a copper grid (diameter, 3.05 mm; 400 meshes; mesh opening size, 37 × 37 μm) for AFM (Digital Instrument with Nanoscope software) and TEM (JEM 2010 with DigitaMicrograph software) observations and also to a Si/SiO_2 _substrate for optical and scanning electron microscopic (SEM) observation. Characterizations are performed on six samples prepared using identical process parameters with a Renishaw Raman microscope (633-nm wavelength).

## Results and Discussion

**Figure 2:**
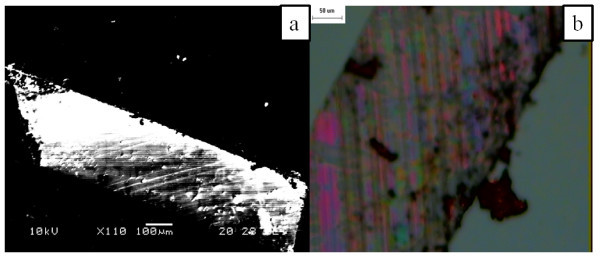
**Images of cleaved layers**. **(a) **SEM image, **(b) **optical microscope image (scale 50 μm).

Under perfect cleaving conditions, we can expect the layer size to be comparable to the dimensions of the face of the pyramid (1 × 0.5 mm). We were able to observe layers with approximate dimensions of 900 × 300-μm area and with thickness range of a few tens of nanometers. The observed layers are shown in Figure 2. The layer dimensions were seen to be approximately 900 × 300-μm area.

During every experimental run, it involved a series of 20 cleaving passes. During every pass, a layer is generated. As the wedge retracts and is ready for the next pass, the layer just generated remains adhered to the wedge surface. The subsequent cut generates another layer which pushes the previous layer further onto the wedge and subsequently on to the water bath. When the new layer goes underneath the previous layer or when the layer reaches the water surface, then in some cases curling of the layers was observed. More often than not a series of layers were observed floating on the water bath. The process is yet to be optimized and the current success rate in cleaving to obtain layers of 900 × 300-μm area is more than 50%.

**Figure 3:**
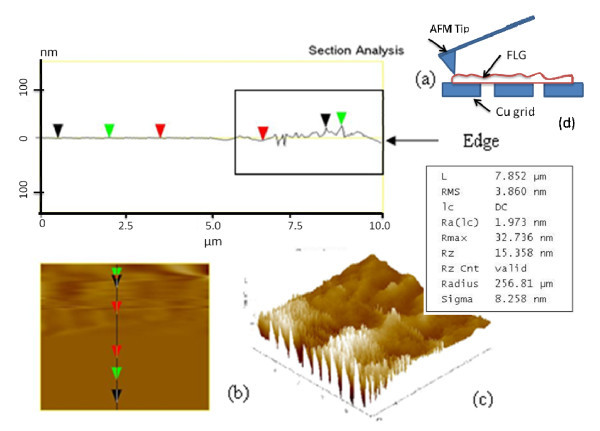
**AFM image**. **(a) **Sectional analysis of edge, **(b) **plan view of edge, **(c) **3-D topography, **(d) **position of AFM tip.

Atomic force microscope operated in the tapping mode is used to determine the thickness of the layers obtained. The sectional analysis (Figure 3) of this data shows that the layer thickness is almost equal to few tens of nanometers. It is also seen that the edges of the layers are composed of uneven thickness as shown in Figure 3a. Figure 3b represents the plan view and Figure 3c shows the topography of a measured area.

**Figure 4:**
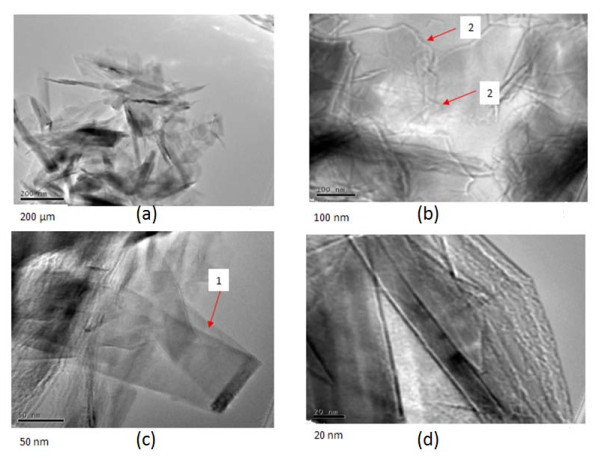
**TEM images**. TEM images without ultrasonic oscillation **(a) **large FLG edges, **(b) **and **(d) **folded FLG, **(c) **large graphene sheet with rolled edge.

**Figure 5:**
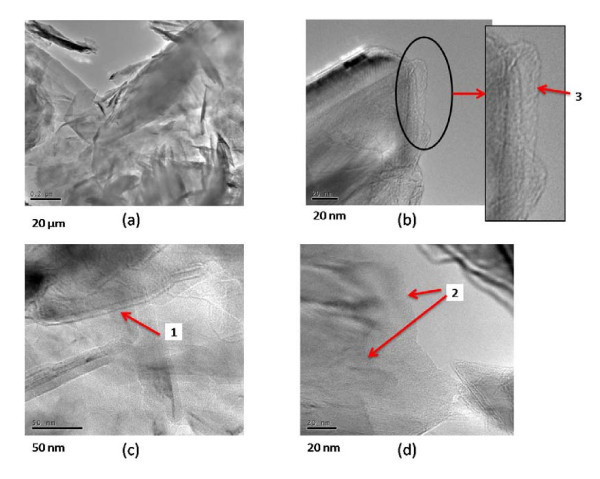
**TEM images**. TEM images with ultrasonic oscillation **(a) **FLG, **(b) **Edge of graphene sheet, **(c) **and **(d) **folded FLG.

Observations using TEM of the few layer graphenes obtained with and without oscillations are shown in Figure 4 and 5 respectively. In the micrographs, of layers without application of oscillations, the folded graphene sheet is clearly visible (marked as 1). In addition, several grain boundaries (marked as 2) are also observed. No other notable edge structures are seen.

Figure 4a shows an area where the sheet appears to be heavily crumpled. In the micrographs of layers obtained with application of oscillations, grain boundaries, folded graphene sheets, and smooth areas of the sheets are also clearly observed. No heavily crumpled regions were seen, but some structures that seem to resemble nanohorns can be observed (marked as 3 in the enlarged area), which needs further investigation. Nanohorns are considered as structures resulting from crushing of a single sheet of graphene [17]. The large surface area of the nanohorns is reported to be useful in various applications such as hydrogen gas storage.

Raman spectroscopy data of the cleaved layers, produced with and without oscillation indicates several features such as the D band (information about defects), G band (in plane vibration) and 2D band (stacking order); these correlated well with reported data in the literature [3,18]. There are no differences in the D band positions (1,332 cm^-1^) with and without oscillations. However, this D band position observed is different from that of bulk graphite (1,355 cm^-1^). The G band position was 1,577 cm^-1 ^with oscillation and 1,578 cm^-1 ^without. The Raman data is further analyzed using a curve fitting method involving deconvolution and fitting two Lorentzian functions, HOPG being a polycrystalline material [19].

**Figure 6:**
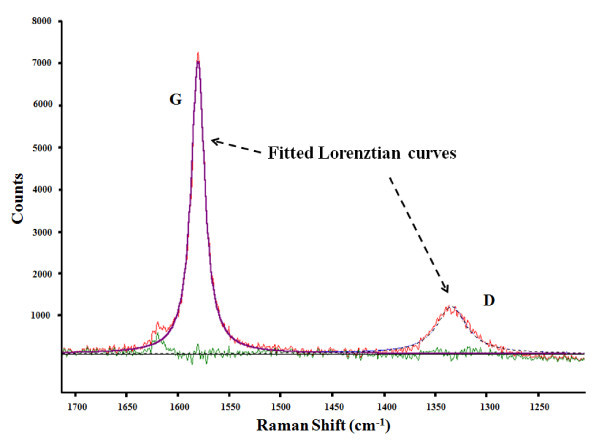
**Lorenztian curve fitting of Raman spectroscopy data (GRAMS wire software)**.

**Figure 7:**
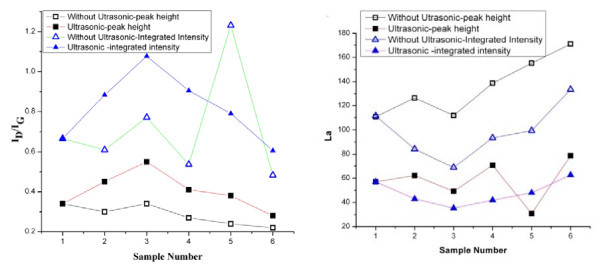
**The values of La calculated using experimentally obtained values of *I***_**D**_***/I***_**G.**_. **(a) ***I*_D_*/I*_G _ratio obtained by both direct peak height measurement and using integrated area method. **(b) **The *La *values calculated using both these methods is plotted

Figure 6 shows the fitted curves obtained, from which the ratio of D and G band peak intensities (*I*_D_*/I*_G_) can be obtained. There are two different ways to calculate this *I*_D_*/I*_G _ratio. One method is to obtain it directly from the peak height [19-22] and the other way is to use the integrated area of the fitted curves [23-25]. Here, both methods are used to analyze the Raman data. The *I*_D_*/I*_G _ratio obtained using both peak height and integrated intensity methods are plotted in Figure 7a for the six samples. A statistical two-sample *t *test conducted on the samples showed that the *I*_D_*/I*_G _ratios for the layers obtained with and without oscillation, calculated using the direct peak height method, were statistically different (*p *value = 0.031 at 95% confidence); thus oscillation has some distinct effect on the process and the layers obtained.

The *I*_D_*/I*_G _ratios can also be used to calculate the crystallite size. The average crystallite size (La) and excitation laser energy both are correlated with the *I*_D_*/I*_G _ratio. There are different equations reported in the literature to estimate La. The original equation determined by Tuinstra and Koenig [26] is said to be not appropriate for all graphite forms. Hence, a general formula for La involving any excitation energy, *E*_*l *_, was proposed by Canado et al. [23] as shown in Equation 1 is adopted here.

(1) $$\text{La}=560\times {\left(I_{D}/I_{G}\right)}^{-1}\times {\left(E_{l}\right)}^{4}$$

The values of La calculated using this equation and experimentally obtained values of *I*_D_*/I*_G _are plotted as shown in Figure 7. It appears that La is smaller when ultrasonic oscillation is applied to the wedge. The value of La is inversely proportional to "amount of crystal boundary" and is a measure of dislocations, vacancies, as well as number of non-graphitic atoms, which in turn is proportional to chemical functionality and shear strength of linkages [22]. The amount of disorder is an indication of fraction *sp*^*2 *^bond and it is a measure of electrical, mechanical, and optical properties. The lower value of La when oscillations are applied indicates the improved the quality of the layers obtained. Also, higher the value of La, lower is said to be the shear strength and from Figure 7 we can conclude that shear strength tends to increase when ultrasonic oscillation used.

## Conclusion and future work

We have demonstrated a novel mechanical cleavage technique to produce few layer graphene from bulk graphite using an ultrasharp diamond wedge assisted by ultrasonic oscillations. AFM measurements indicate that the proposed mechanical cleaving method is capable of producing thin layer graphene with a thickness of tens of nanometers. TEM studies reveal that there is considerable amount of attention required to understand the edge formation with ultrasonic oscillation usage since structures that seem to resemble nanohorns were observed. Application of ultrasonic vibrations along the tool edge is seen to significantly reduce the *I*_D_/*I*_G _ratios seen in a Raman spectrum. Hence, the applied oscillations may have potential to reduce the defects in cleaved layers. The application of ultrasonic vibration also reduces the crystallite size. In the future we will perform molecular dynamic simulations to understand the cleavage mechanism and the effect of process parameters on the cleavage.

## Competing interests

The authors declare that they have no competing interests.

## Authors' contributions

BJ designed and conducted all experiments and characterisation and helped in drafting the manuscript. SS conceived of the study, participated in the experimental setup design, and drafted the manuscript. Both BJ and SS have read and approved the final manuscript.
